# Seafloor vegetation map of man-made boulders reef by underwater photogrammetry: Suggestions for site selections in macroalgal bed creations

**DOI:** 10.1371/journal.pone.0341865

**Published:** 2026-03-02

**Authors:** Takayuki Kanki, Wataru Sano, Masami Sannoh, Hironobu Kan

**Affiliations:** 1 Faculty of Engineering, Kyushu University, Fukuoka-shi, Fukuoka, Japan; 2 Faculty of Social and Cultural Studies, Kyushu University, Fukuoka-shi, Fukuoka, Japan; 3 Special Researcher (PD), Japan Society for the Promotion of Science, Tokyo, Japan; 4 Okayama University School of Education, Kita-ku, Okayama-shi, Okayama, Japan; 5 Research Center for Coastal Seafloor, Kyushu University, Fukuoka-shi, Fukuoka, Japan; IEAPM: Instituto de Estudos do Mar Almirante Paulo Moreira, BRAZIL

## Abstract

Macroalgal beds play important roles in coastal ecosystems by supplying oxygen, providing food resources, and offering habitats for various marine animals. In recent years, macroalgal beds have significantly declined due to various factors, leading to efforts toward macroalgal bed restorations. Despite these efforts, suitable sites for macroalgal bed creations remain insufficiently understood. In this study, we investigated suitable location conditions for establishment of macroalgal beds by mapping the seafloor vegetation. In an 80 m × 20 m area at a depth of 4.5–9.2 meters in Himeshima Island, Fukuoka, Japan, man-made boulders reef was created by the local government in the fiscal year 2016 for macroalgal bed creation. A 3D model of the seafloor, with a resolution of 0.07 meters, was created using underwater photogrammetry, enabling the visualization of macroalgal vegetation on these artificial boulders through a detailed seafloor vegetation map. Rich macroalgal vegetation, including *Sargassum* spp. and *Undaria pinnatifida* was established on the boulders placed on sandy bottoms. In contrast, on the boulders placed on natural cobble/boulder reefs, only a short-lived seaweed *Colpomenia sinuosa* was observed, and almost all boulders showed no vegetation. Even on the sandy bottoms, boulders that were adjacent to or surrounded by natural cobble/boulder reefs, or those that were piled together, tended to have poor vegetation. For effective macroalgal beds creation, installation of boulders at a low density on sandy bottoms would be preferable, while avoiding the areas on or adjacent to natural cobble/boulder reefs.

## 1 Introduction

Macroalgal beds play an important role in coastal ecosystems by producing oxygen and food resources through primary production [1,2]. They are also hot spots of biodiversity, supplying habitats for various animals including commercially important fishery species. Sea urchins, abalones, and snails consume seaweeds as a food resource [3–5], and a squid *Sepioteuthis lessoniana* spawns on macroalgal beds [6]. Many small invertebrates, such as Porifera, Bryozoa, Cnidaria, and Crustacea inhabit on macroalgal beds [7–10], some of these invertebrates are also important food resources for carnivorous fish [11,12]. Additionally, macroalgal beds serve as nurseries, offer refuge from predators, and provide food resources for various juvenile coastal fish [13–16]. Drifting seaweeds mainly from *Sargassum* species also function as nurseries for juvenile pelagic fish, including commercially important fishery species such as yellowtail *Seriola quinqueradiata*, jack mackerel *Trachurus japonicus*, and greater amberjack *Seriola dumerili* [17–19].

In recent years, macroalgal beds in temperate waters have significantly declined worldwide [20–22]. Decline of macroalgal beds is attributed to various factors, including the pollution and increased turbidity due to urbanization [23,24], sediment accumulation [25,26], reduced growth rates or withering caused by rising seawater temperatures [27], and acidification [28]. In temperate waters, grazing pressure of seaweeds from herbivorous animals such as sea urchins, snails, and herbivorous fish is considered one of the most critical factors contributing to decline of macroalgal beds [21,29–31]. Grazing pressure by herbivorous animals have increased due to rising seawater temperatures [32,33], the overfishing of species that prey on these herbivores [34], and arrival of new herbivorous species due to tropicalization [35].

Various methods have been trialed for restoring macroalgal beds, including reducing the grazing pressure by removing of sea urchins and herbivorous fish [36–38], setting up marine protected areas for reestablishment of predatory interactions [39–41], or using protective cages to shield macroalgae from herbivorous animals [30,31,42,43]. Additionally, techniques artificially supplying mature plants or fertilized eggs by direct transplantation or spore-bag method [42–44], and installing new substratum for promoting recruitments of macroalgae [45–47] are commonly employed. Man-made reefs constructed from natural boulders or concrete blocks are frequently installed to also serve as fishing reefs or wave-dissipating structures, in addition to their role as macroalgal bed creation.

Methods for restoration of macroalgal beds by installing new substratum have been explored, focusing on factors such as substratum relief [48 48], installation season [49] and vegetative succession post-installation [50,51]. Effective macroalgal bed creations by substratum installation requires appropriate site selection based on the current knowledge of the environmental conditions suitable for macroalgal beds establishment. For example, seaweed biomass on artificial reefs installed on rocky reefs was lower due to grazing by sea urchins, compared to those on sandy bottoms [45,50]. In addition, water motion can inhibit feeding by herbivorous species [52,53]. However, there are still few studies on mapping the seascape and macroalgal vegetation to discuss detailed habitat suitable conditions for macroalgal vegetation, likely due to limitation in technology for mapping detailed seafloor topography and vegetation simultaneously.

Photogrammetry is a technique for reconstructing three-dimensional structures of objects or scenes using photographs taken from multiple viewpoints, such as Structure from Motion [54]. It has been widely used for topographic mapping, with numerous open-source and commercial software available [55–57] that allow non-professional users to easily create 3D models. Since 2010s, underwater photogrammetry has seen significant advancements for capturing the three-dimensional characteristics of seafloor topography and underwater structures [57–83].

Creating a 3D model of seafloor topography using underwater photogrammetry enables the collection of data on the distribution of benthic organisms [75,77,79,82], detailed topography of seafloor or artificial reef [78], metrics of benthic organisms [84,85], and geomorphic indicators [67,70,72,77]. The quantitative information on habitat and seafloor topography from underwater photogrammetry can be utilized to elucidate suitable habitat conditions and estimate habitat map for benthic organisms, including seaweeds [76,77].

For investigating spatial distribution of seafloor vegetation, various methods have been employed, including multispectral images by UAVs [86,87], satellite images [88], multi-beam echosounders [89,90], and side-scan sonar [91]. Photogrammetry methods can use the photographs to not only reconstruct the 3D models but also identify seaweed species and their distributions. Consequently, underwater photogrammetry is considered the most suitable method for creating detailed seafloor vegetation maps, particularly on subtidal rocky shores with complex topography.

In this study, we utilized underwater photogrammetry to map the detailed seafloor topography of an 80 m × 20 m area of man-made boulders reef. In addition, we mapped the seafloor vegetation within this area. We analyzed how seafloor vegetation varied with bottom type and boulder placements. Based on these analyses, we discussed the suitable and unsuitable locational conditions for establishment of macroalgal beds and provided suggestions for effectively macroalgal bed creation by boulder installation.

## 2 Materials and methods

### 2.1 Study site

**Fig 1:**
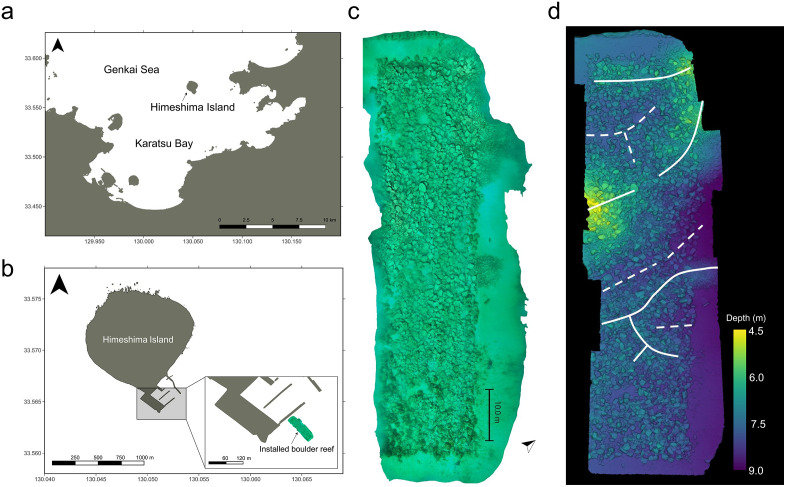
Location of the study site and 3D model of the artificial boulder reef. **(a)** Location of the study site and **(b)** the man-made boulders reef in Himeshima island. These maps were created by editing public domain, open access base data from the Geospatial Information Authority of Japan (GSI). **(c)** Orthomosaic image and **(d)** depth map of the total man-made boulders reef in the study site. The upper side of the 3D model in Figs 1c, d is the shore side, and the lower side is the offshore side. The left-edge areas in the 3D models failed to create 3D constructions because of a lack of photographs. In Fig 1d, solid lines indicate the natural cobble/boulder ridges, and dotted lines indicate the valleys surrounded by the natural cobble/boulder reef.

The study site was located in the southern part of the Himeshima Island (33.564°N, 130.052°E), in Fukuoka Prefecture, Japan (Fig 1a). Himeshima Island is located on the mouth of Karatsu Bay. The average monthly water temperature in Karatsu Bay is a maximum of 26.9°C, and a minimum of 10.2°C in a typical year [92].

In the fiscal year 2016, artificial boulders were installed on the seafloor in areas covering an area of 80 m × 20 m for the purpose of creating artificial macroalgal beds and increasing fisheries resources such as sea urchins, abalones, and turban shells by the Fisheries Promotion Division of Fukuoka prefecture, Japan (Fig 1b, 1c, 1d). These boulders, made from natural rock and cut to a diameter of approximately 1 meter, were placed at depths of 4.5–9.2 meters and surrounded by sandy seafloor (Fig 1c, 1d). The area featured natural cobbles and boulders that formed several ridges (Fig 1d), with the artificial boulders were placed across these cobble/boulder ridges and sandy bottoms.

### 2.2 Underwater photogrammetry

#### 2.2.1 Field survey.

**Fig 2:**
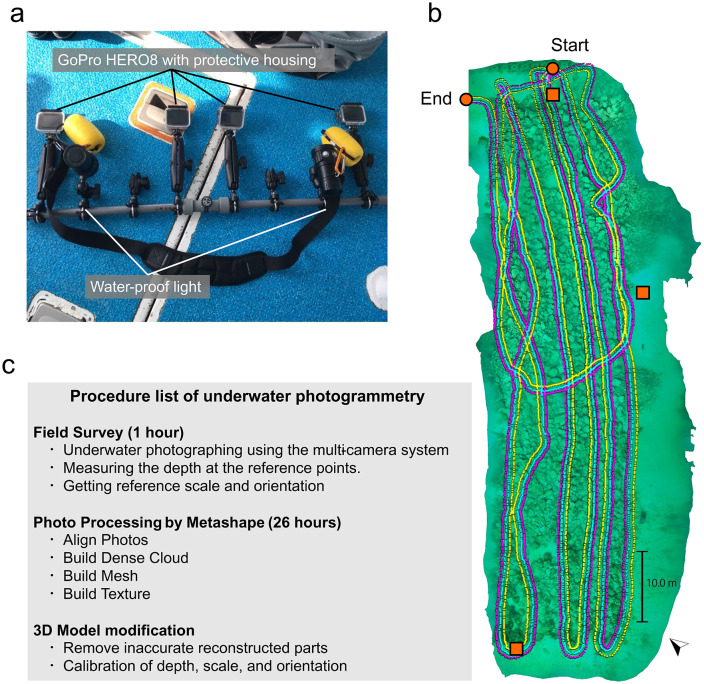
Schematic diagram of field survey and underwater photogrammetry. **(a)** Multi-camera system (MURAKUMO HANDY) used in photographing for underwater photogrammetry. One of four cameras is a spare one. **(b)** Camera trails estimated in photogrammetry processing in Metashape. Each pink, yellow, and cyan plot indicate the trail of each camera. Two orange circles show start and end points of photographing. Three orange squares indicate reference points for depth. **(c)** Flow diagram illustrating the creation of the 3D model through underwater photogrammetry.

An underwater photographing survey was conducted on March 7, 2023, using a multi-camera system equipped with three GoPro HERO8 units and two waterproof lights (Fig 2a). This system, the MURAKUMO HANDY, was cooperatively developed by the World Scan Project and Research Center for Coastal Seafloor at Kyushu University. The survey was conducted by verbal agreement by Himeshima branch, a fishery cooperative in Itoshima.

In photogrammetry, a 60–80% overlap of the photographs is required to reconstruct high-quality 3D models [93–95]. Achieving sufficient overlap is difficult in underwater photography by a single camera without navigation. However, using a multi-camera system, by adjusting the camera position so that the images between adjacent cameras are overlapped, sufficient overlap can be achieved efficiently. For this survey, the time-lapse function was utilized, capturing photographs at one-second intervals. An 80 m × 20 m area was scanned using SCUBA diving, with tracks shown in Fig 2b. The cameras were oriented downward and maintained approximately 2.0 m above the seafloor. A total of 9,751 shots were taken over 57 minutes.

#### 2.2.2 3D model reconstruction.

Subsequently, the acquired photos were processed using Metashape 1.8.4 (Agisoft) to generate a 3D model of the seafloor topography. In Metashape, the photos were imported, and the tools “Align Photos”, “Build Dense Cloud”, “Build Mesh”, and “Build Texture” were applied (Fig 2c). In “Align photos”, “Accuracy” was setting high. The total computation time in Metashape was approximately 26 hours, and used up to 18.59GB of memory. The resolution of 3D model was reduced in Metashape, and its scale, orientation, and depth were adjusted using Meshlab [96] and Julia 1.5.3 (S1 Text). The final 3D model, created through underwater photogrammetry, covered a horizontal area of 2,507 m^2^, with an average vertex resolution of 7.9 cm.

### 2.3 Investigating seafloor vegetation and bottom type

The surfaces of the artificial boulders were photographed with the waterproof digital camera OLYMPUS tough TG-6, in addition to GoPro HERO8 photos, as the GoPro photo’s resolution was sometimes insufficient for identifying seaweed species. The OLYMPUS tough TG-6, equipped with two water proof lights, was used for identifying seaweed species on March 7, 2023, simultaneously with the underwater photogrammetry survey.

**Fig 3:**
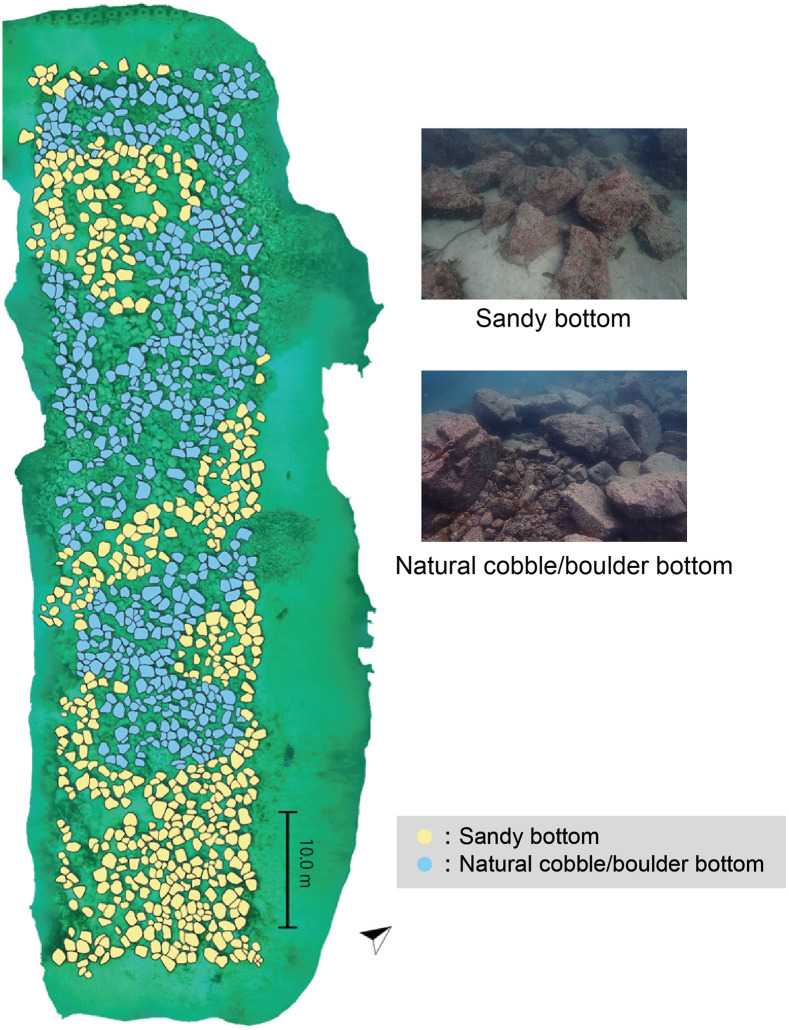
Bottom type of the artificial boulders. Filled yellow boulders indicate the artificial boulder on the sandy bottom and filled blue boulders indicate the natural cobble/boulder bottom. A total of 550 artificial boulders were placed on the sandy bottoms, and 561 on the natural cobble/boulder bottom. The lower part of the image represents the offshore side.

March is the season when seaweed diversity is highest and most large seaweeds begin to elongate. Based on the photos and the 3D model, we identified and categorized the macroalgal vegetation and bottom type of 1,111 artificial boulders, excluding some boulders that could not be assessed due to unclear photos or because they were outside the 3D model’s coverage. Of these boulders, 550 were located on the sandy bottom and 561 were on the natural cobble/boulder bottoms (Fig 3).

The presence or absence of medium-to-large seaweeds on each boulder, including *Undaria pinnatifida*, *Sargassum horneri*, perennial *Sargassum* spp., *Colpomenia sinuosa*, and geniculate coralline red algae was determined from the photographs (S2 Fig). *S*. *horneri* was distinguishable from other perennial *Sargassum* spp. by its spindle-shaped air bladders. The perennial *Sargassum* spp. include *S. patens*, *S. macrocarpum* and *S. yamamotoi*. Additionally, on May 26, 2023, medium-to-large seaweeds on the artificial boulders were sampled via SCUBA diving, and identified in the laboratory (Table 1).

**Table 1:** A list of medium to large sized seaweeds hand-collected on May 26, 2023. Fourteen species of seaweed were collected on May 26, 2023. *Colpomenia sinuosa* was observed on March 7, but not on May 26 survey.

Order	Species
Codiales	*Codium fragile*
	*Codium subtubulosum*
Dictyotales	*Dictyopteris undulata*
	*Zonaria diesingiana*
	*Padina arborescens*
Scytosiphonales	*Hydroclathrus clathratus*
	*Colpomenia sinuosa*
Laminariales	*Ecklonia cava* ssp. *kurome*
	*Undaria pinnatifida*
Fucales	*Sargassum horneri*
	*Sargassum patens*
	*Sargassum yamamotoi*
	*Sargassum macrocarpum*
Rhodymeniales	*Coelarthrum* opuntia
Corallinales	*Amphiroa dilatata*

The vegetation types and vegetation scores were categorized as follows: bare rock (score 0), *C*. *sinuosa* community (score 1), coralline red algae community (score 2), *U*. *pinnatifida* community (score 3), *S. horneri* community (score 4), Sargassum community (score 5), and perennial *Sargassum* spp. and *U*. *pinnatifida* communities (score 6) (Table 2). Vegetation scores were established based on their maintenance period, algal body length, and species diversity of the macroalgal beds. *C. sinuosa* is a very short-lived species that attaches only for a short period in spring, most of which disappears from April to May; that is, the *C*. *sinuosa* community shows bare rocks over a long period, from early summer to winter, with a poor macroalgal bed community. The geniculate coralline red algal community provides habitat for various small invertebrates, but is of low nutrient value for herbivorous animals due to their calcified hard structure [97]. *U*. *pinnatifida* and *S. horneri* are annual species that detach from May to June. The *S*. *horneri* community was assigned a higher vegetation score because *S. horneri* grows to more than 2 meters in length, compared to *U*. *pinnatifida*, which typically grow to approximately 50 cm. The extension period of perennial *Sargassum* spp. extends into June or July, after *U*. *pinnatifida* and *S. horneri* detached, and were assigned a high score. The Sargassum and *U*. *pinnatifida* community was assigned the highest score due to the complex structure of the macroalgal beds and the long extension period. This community consists of medium-sized *U*. *pinnatifida* and large-sized perennial *Sargassum* spp., forming a hierarchical structure.

**Table 2:** Vegetation categorization criteria and scoresheet of the macroalgal vegetation. Seafloor vegetation types and vegetation scores were categorized based on the presence of each seaweed species as follows: *Sargassum* and *U*. *pinnatifida* community (score 6), Sargassum community (score 5), *S. horneri* community (score 4), *U*. pinnatifida community (score 3), coralline red algae community (score 2), *C*. *sinuosa* community (score 1), and bare rock (score 0). In the table, “+” indicates the species were present, “−” indicates absent, and “+/−” indicates that the species wase either present or absent.

*U*. *pinnatifida*	Perennial *Sargassum* spp.	*S. horneri*	*C*. *sinuosa*	geniculate coralline red algae	Score	Vegetation Type
+	+	+/−	+/−	+/−	6	*Sargassum* and *U. pinnatifida* community
−	+	+/−	+/−	+/−	5	*Sargassum* community
+/−	−	+	+/−	+/−	4	*S. horneri* community
+	−	−	+/−	+/−	3	*U. pinnatifida* community
−	−	−	+/−	+	2	Coralline red algae community
−	−	−	+	−	1	*C. sinuosa* community
−	−	−	−	−	0	Bare rock

### 2.4 Geomorphic indicators

For investigating suitable habitat condition, geomorphic indicators (relative height and surface complexity) were computed from the 3D model of seafloor topography (S3 Fig) using our own making source codes with Julia version 1.5.3, as detailed in Supporting information (S4, S6 Figs, S5, S7 Texts). The geomorphic indicators were computed for 1,092 boulders of the total 1,111 artificial boulders. The remaining 19 boulders were located at the edge of the 3D model, so their geomorphic indicators were not computed. The geomorphic indicators for each boulder were adopted the values computed at the summit of each boulder.

### 2.5 Statistical analyses

The effects of the bottom type or geomorphic indicators on the vegetation scores were tested using Welch’s t-test. Pearson’s correlation coefficients between vegetation scores and geomorphic indicators (relative height and surface complexity) were computed for each bottom type. Statistical analyses were performed with R version 4.2.0.

## 3 Results

### 3.1 Presence of seaweed species on the artificial boulders reef

**Fig 4:**
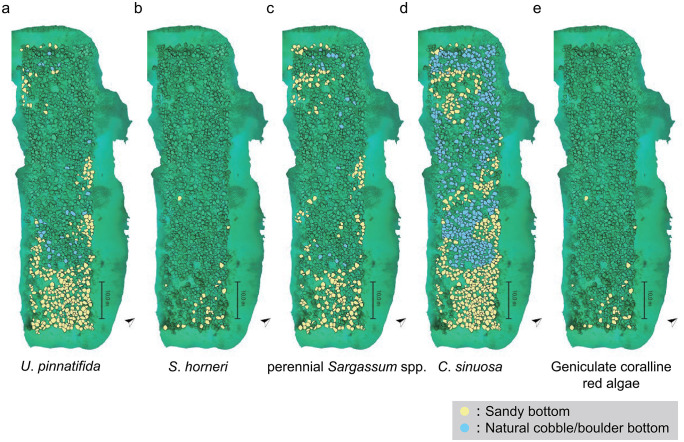
Distribution of each seaweed species on the man-made boulders reef. **(a)** *U*. *pinnatifida*, **(b)** *Sargassum horneri*, **(c)** perennial *Sargassum* spp., **(d)** *C*. *sinuosa*, and **(e)** geniculate coralline red algae. The lower sides in the maps represent the offshore side. Filled yellow boulders indicate the artificial boulders on the sandy bottoms and filled blue boulders indicate on the natural cobble/boulder bottom. *C. sinuosa* was present throughout the man-made boulders reef, but other species tended to be present on sandy bottom in the offshoreside.

Most seaweed species were present predominantly on the boulders on sandy bottom, but *C. sinuosa* was present on the boulders of any bottom type.. Among the 1,111 artificial boulders, the most abundant species was *C. sinuosa*, found on 883 boulders (79.5%). *Undaria pinnatifida*, perennial *Sargassum* spp., geniculate coralline red algae, and *S. horneri* were present on 353 (31.8%), 243 (21.9%), 151 (13.6%), and 32 (2.9%) boulders, respectively. On the sandy bottom, *C. sinuosa* was found on 436 (79.3%), *U. pinnatifida* on 321 (58.4%), perennial *Sargassum* spp. on 218 (39.6%), geniculate coralline red algae on 145 (26.4%), and *S*. *horneri* on 31 (5.6%) boulders. On the natural cobble/boulder bottom, *C. sinuosa* was present on 447 boulders (79.7%), *U. pinnatifida* on 32 (5.7%), perennial *Sargassum* spp. on 25 (4.5%), geniculate coralline red algae on 6 (1.1%), and *S*. *horneri* on 1 (0.2%) boulders (Fig 4, Table 3, S8 Data).

**Table 3:** Table of the number of artificial boulders with each seaweed species presence, vegetation type, and bottom type (on the sandy bottom and on the natural cobble/boulder bottom). In the table, “+” indicates the species were present, blank indicates absent, respectively.

*U*. *pinnatifida*	Perennial *Sargassum* spp.	*S*. *horneri*	*C*. *sinuosa*	Geniculate coralline red algae	The Number of ABs (On sand/ cobble/ total)	Vegetation Type	The Number of ABs (On sand/ cobble/ total)
+	+	+	+	+	12/ 1/ 13	*Sargassum* and *U. pinnatifida* community (Score: 6)	176/ 5/ 181
+	+	+	+		2/ 0/ 2		
+	+		+	+	82/ 1/ 83		
+	+		+		49/ 4/ 53		
+	+			+	11/ 0/ 1		
+	+				19/ 1/ 20		
	+	+			0/ 1/ 1	*Sargassum* community (Score: 5)	42/ 17/ 59
	+		+	+	1/ 0/ 1		
	+		+		21/ 16/ 37		
	+				19/ 1/ 20		
+		+	+	+	5/ 0/ 5	*S. horneri* community (Score: 4)	15/ 1/ 16
+		+	+		9/ 0/ 9		
		+	+		1/ 0/ 1		
		+			0/ 1/ 0		
+			+	+	24/ 2/ 26	*U. pinnatifida* community (Score: 3)	131/ 24/ 155
+			+		87/ 21/ 108		
+				+	5/ 0/ 5		
+					15/ 1/ 16		
				+	1/ 0/ 1	Coralline red algae community (Score: 2)	4/ 2/ 6
			+	+	3/ 1/ 4		
			+		138/ 397/ 535	*C. sinuosa* community (Score: 1)	138/ 397/ 535
					44/ 108/ 152	Bare rock (Score: 0)	44/ 108/ 152

*U. pinnatifida*, *S*. *horneri*, perennial *Sargassum* spp., and geniculate coralline red algae were primarily present on the sandy bottoms offshore (low side in Fig 4a, 4c, 4e). Notably, *S*. *horneri* and geniculate coralline red algae were absent on the shoreside areas (Figs 4b, 4e). In contrast, *C. sinuosa* was present throughout the artificial boulder reef, both onshore and offshore (Fig 4d).

On May 26, 2023, all species of medium-to-large size seaweeds were hand-collected from artificial boulders at the study site and identified in the laboratory. Fourteen species of medium-to-large size seaweeds were collected (Table 1). A short-lived species, *C*. *sinuosa* was observed on March 7 but not at all on May 26.

### 3.2 Vegetation type on the artificial boulders reef

On sandy bottom, rich vegetation composed of *Sargassum* species and *U*. *pinnatifida* was established on more than half of the boulders. In contrast, on natural cobble/boulder bottom, poor vegetation such as *C*. *sinuosa* community or bare rock was observed on over 90% of the boulders, and the occurrence of rich vegetation was extremely low.

**Fig 5:**
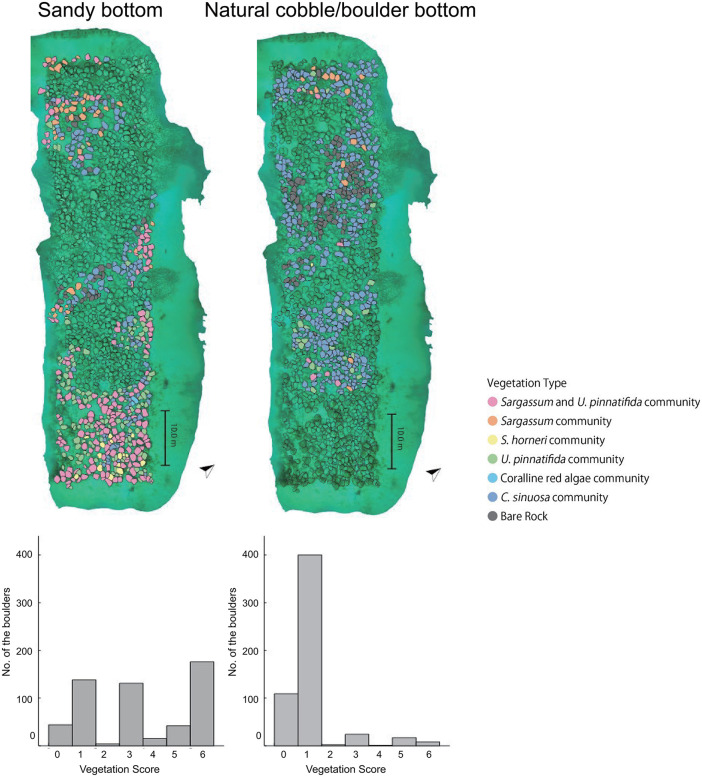
Macroalgal vegetation map and histograms of vegetation scores on the sandy bottom and on the natural cobble/boulder bottom. The vegetation scores tended to be higher on sandy bottom, and lower on the natural cobble/boulder bottom. The lower sides of the vegetation maps represent the offshore sides.

Among the 1,111 artificial boulders surveyed, the most abundant vegetation type was *C*. *sinuosa* community, found on 535 artificial boulders (48.2%). *Sargassum* and *U*. *pinnatifida* community, *U*. *pinnatifida* community, *Sargassum* community, *S*. *horneri* community, and coralline red algae community were present on 181 (16.3%), 155 (14.0%), 59 (5.3%), 16 (1.4%), and 6 (0.5%) boulders, while 153 boulders (13.8%) were bare rock. On the sandy bottom, the most abundant vegetation type was *Sargassum* and *U*. *pinnatifida* community, which was present on 176 artificial boulders (32.0%). *C*. *sinuosa* community, *U*. *pinnatifida* community, *Sargassum* community, bare rock, *S*. *horneri* community, and coralline red algae community were present on 138(25.1%), 131 (23.8%), 44 (8.0%), 42 (7.6%), 15 (2.7%), and 4 (0.7%) boulders, respectively. On the natural cobble/boulder bottom, the most abundant vegetation type was the *C*. *sinuosa* community, which was present on 397 artificial boulders (70.8%). Bare rock, *U*. *pinnatifida* community, *Sargassum* community, *Sargassum* and *U*. *pinnatifida* community, coralline red algae community, and *S*. *horneri* community were present in 108 (19.3%), 24 (4.3%), 17 (3.0%), 5 (0.9%), 2 (0.4%), and 1 (0.18%) boulders, respectively (Fig 5, Table 3, S8 Data). The vegetation scores of artificial boulders on sandy bottoms were high, whereas those on natural cobble/boulder bottom were low; the average scores were 3.4 and 1.1, respectively (Fig 5, Welch’s t-test, *p* < 0.001).

### 3.3 Seafloor vegetation and geomorphic indicators

**Fig 6:**
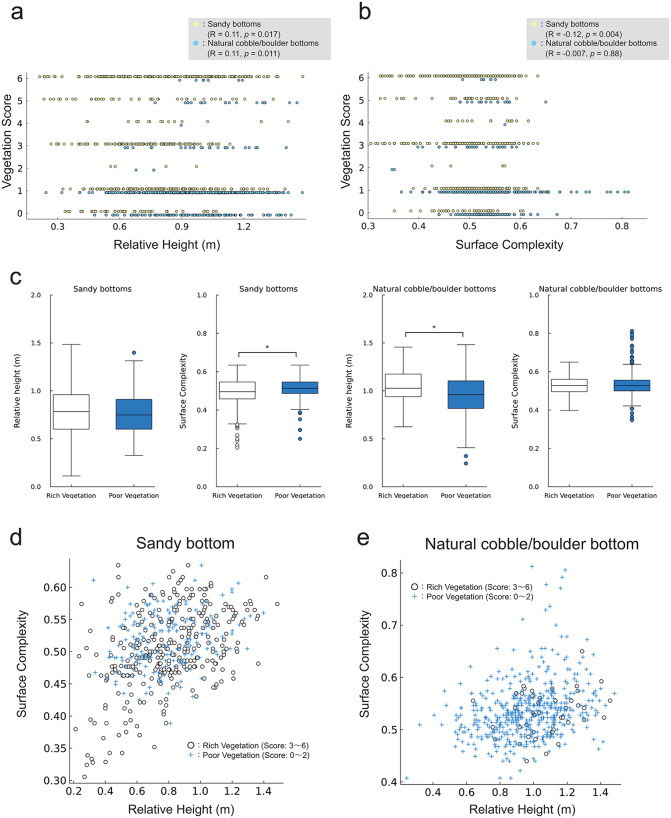
Vegetation scores for bottom type and geomorphic indicators. Plots of **(a)** vegetation scores and relative height, **(b)** vegetation scores and surface complexity on sandy bottom and natural cobble/boulder bottom. In these plots, the vegetation scores were shown by adding +0.1 on sandy bottom and −0.1 on natural cobble/boulder bottom for visualization. **(c)** Boxplots of geomorphic indicators with bottom type and vegetation richness. The box plots display the median (central line), the interquartile range (box), and the whiskers extending to the most extreme data points not considered outliers. Outliers are shown as individual dots. Asterisks denote statistically significant differences (*p* < 0.01, t-test). Plots of relative height and surface complexity **(d)** on sandy bottom, and **(e)** on natural cobble/boulder bottom.

**Fig 7:**
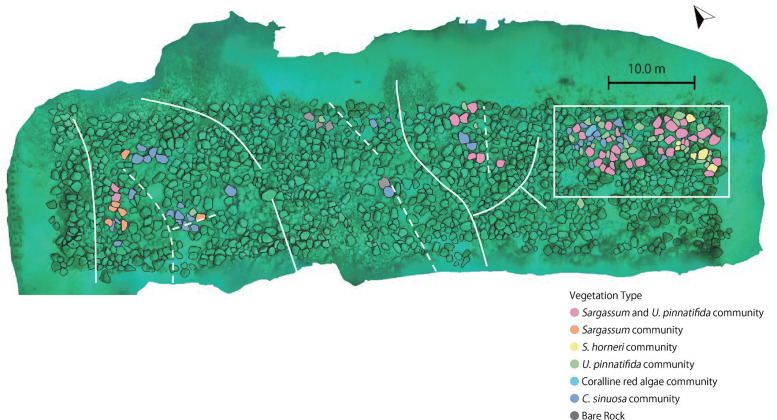
Macroalgal vegetation of the boulders on the sandy bottoms and high surface complexity (> 0.55). Dotted lines indicate the valley surrounded by the natural cobble/boulder reef (as in Fig 1d), and solid lines indicate the natural cobble/boulder ridges. Low vegetation scores *C*. *sinuosa* community and bare rocks were observed on the artificial boulders on the sandy bottoms in the valley surrounded by cobble reefs (across the dotted lines). Artificial boulders were piled in a white square. Most of the artificial boulders piled on the sandy bottoms exhibited high vegetation scores, though some had low score vegetation *C*. *sinuosa* community. The right side of the image represents the offshore side.

Even on the sandy bottoms, poor vegetation (*C*. *sinuosa* community and bare rock) tended to establish on high surface complexity (Figs 6, 7), which corresponded to the boulders located at the end of the valley surrounded by cobble ridges or adjacent to cobbles, where the artificial boulders were piled (S3c Fig). In addition, on the natural cobble/boulder bottoms, rich vegetation tended to establish on high relative height (Fig 6).

Between relative height, complexity and vegetation scores, weak negative correlations of –0.12 (*p* < 0.001) and –0.193 (*p* < 0.001) were observed. On the sandy bottoms, the correlation coefficients were 0.11 (*p* = 0.017) and –0.12 (*p* = 0.005), respectively (Figs 6a, 6b). On the natural cobble/boulder bottom, the correlation coefficients were 0.11 (*p* = 0.011) and –0.007 (*p* = 0.88), respectively (Figs 6a, 6b).

Integrating vegetation scores greater than 3 as rich vegetation, and less than 2 as poor vegetation, relative height on cobble bottom and surface complexity on sandy bottom had significant effects (t-test, *p* < 0.001, *p* = 0.003) (Fig 6c). Relative height on sandy bottom and surface complexity on cobble bottom had no significant effects (t-test, *p* = 0.58, 0.66) (Fig 6c). On the sandy bottom, on the boulders with surface complexity less than 0.4, rich vegetation established. In such conditions, relative height was also small (Fig 6d). On the natural cobble/boulder bottom, on the boulders with relative height less than 0.75, or with surface complexity greater than 0.60, poor vegetation was dominant (Fig 6e).

## 4 Discussion

### 4.1 Suitable conditions for establishment of macroalgal beds

On most of the artificial boulders placed on the natural cobble/boulder bottom, only the short-lived species *Colpomenia sinuosa* was growing or bare rocks. However, on the sandy bottoms, various seaweeds *Undaria pinnatifida*, *Sargassum horneri*, perennial *Sargassum* spp., and geniculate coralline red algae were present on the artificial boulders. Previous study reported that higher biomass and diversity of seaweeds were present on concrete blocks set on sandy bottoms rather than on rocky bottoms [45], which is consistent with the results of this study.

Rich vegetation established on boulders subjected to strong sandy effects (sand sedimentation or friction from sand drift), whereas poor vegetation established on boulders subjected to low sandy effects. Even on sandy bottom, on the piled boulders, where sandy effect was low, with position elevated from sandy seafloor. Additionally, in valleys surrounded by cobble/boulder ridges, which were sheltered from external flow and waves, sandy effects were reduced. Consequently, poor vegetation established in these locations, similar to that observed on natural cobble/boulder bottom.

On boulders exposed to strong sandy effects, grazing pressure by herbivorous animals was considered lower than on natural cobble/boulder bottom. *Heliocidaris crassispina* is one of the most active seaweed grazers in temperate waters [98], and many individuals were also observed in coastal areas of Himeshima Island (S9 Fig). *H*. *crassipina* tend to avoid areas subjected to sandy effects [98], therefore, grazing pressure by *H*. *crassipina* was considered lower on boulders with strong sandy effects, facilitating the establishment of rich vegetation. A decrease in grazing pressure due to sedimentation has also been reported for limpets and other sea urchin species [99–102]. In addition, the spaces between piled boulders created shaded areas that provided shelter for grazing animals, whereas a single boulder on sandy bottom did not offer such space (S3 Fig).

Many *H*. *crassipina* were observed on the natural cobble/boulder reef continuous with the study site, but few *H*. *crassipina* were observed on the artificial boulder reef in this snapshot survey conducted on March 2023. At the study site, *H*. *crassipina* are active only during early spring (S9 Fig), and they may migrate to the artificial boulder reef to feed on the seaweeds during this period. In addition, not only sea urchins but also snails and limpets which prefer rock with less sand effects may serve as important grazer as in other temperate regions [99,101,102].

Sand sedimentation can have both positive and negative effects on seaweed distribution. At the study site, moderate sand sedimentation and wave exposure created conditions that supported low grazing pressure, suggesting a suitable environment for the establishment of macroalgal beds. The recruitment and growth of some seaweed species are inhibited by sand sedimentation or friction from drifting sand [103–106]. In addition, under strong sand sedimentation, the boulders would become completely buried. In contrast, friction from drifting sand can remove already established sessile organisms, thereby providing new substrates for seaweed recruitment [107]. Sand sedimentation did not provide new substrates in areas lacking habitat substrate for seaweeds, since most of the boulders on cobble bottom were bare rock. The primary factor is likely the reduction of the grazing pressure although it is possible that microscopic stages of seaweeds may preferentially settle on boulders affected by sand sedimentation. Information on settlement preferences of seaweeds is limited, with only a few species, such as *Porphyra umbilicalis*, known to its filamentous boring into shell [108]. In rocky shore ecosystem, sand sedimentations play an important role in shaping benthic communities; however, the mechanisms and details are not yet fully understood [107], and further research is needed.

*U*. *pinnatifida*, *S*. *horneri*, and geniculate coralline red algae tended to be present on the artificial boulders on the sandy bottoms, particularly at the offshore end. This may be due to the presence of natural pebbles on the offshore side of these sandy areas, where these species thrived, facilitating the attachments of spores and embryos from the matured seaweeds. In addition, extensive natural macroalgal beds located further offshore may have contributed to the recruitment of these seaweeds. Nutrient flux, a product of nutrient concentration and flow velocity, is known to be crucial for seaweed growth [109–111]. The artificial boulders at the offshore end were exposed to high flow velocity and nutrient flux, probably making them suitable for the growth of the annual large species *U*. *pinnatifida* and *S*. *horneri* which require rapid growth speed.

On the natural cobble/boulder bottoms, high score vegetations (*Sargassum* community (score 5), *Sargassum* and *U*. *pinnatifida* community (score 6)), were established only at locations with high relative height, most of which exceeded 0.8 m (Fig 6a). *U*. *pinnatifida* was frequently observed on the natural cobble/boulder bottoms on the offshore side and near sandy areas (Fig 4). These offshore or elevated locations may be exposed to higher flow velocity, which could enhance nutrient fluxes, promote rapid growth in seaweeds, and reduce feeding pressure from herbivorous animals [53]. Although specific flow velocities at these sites were not measured, detailed information on seafloor topography would be valuable for accurately simulating environmental fluid dynamics [112]. In the future, comprehensive analyses integrating seafloor topography, physical environmental factors such as fluid dynamics and light intensity, and seafloor vegetation may become possible. An evergreen kelp *Ecklonia radicosa* subsp. *kurome*, a common species in temperate macroalgal bed ecosystems and on the natural macroalgal beds around the study site, was rarely observed on the man-made boulders reef. Its scarcity on the man-made boulders reef was likely due to the short time since their installation. In the same genus as *E*. *cava*, the dispersal distance of spores was reported to be around 2 meters from the thallus [113], the dispersal ability of *E*. *radicosa* subsp. *kurome* was considered also low. This limited dispersal ability contrasts with that of *Sargassum* species, which can spread over long distances via drifting seaweeds [114].

### 4.2 Suggestions for site selection for macroalgal beds creation

**Fig 8:**
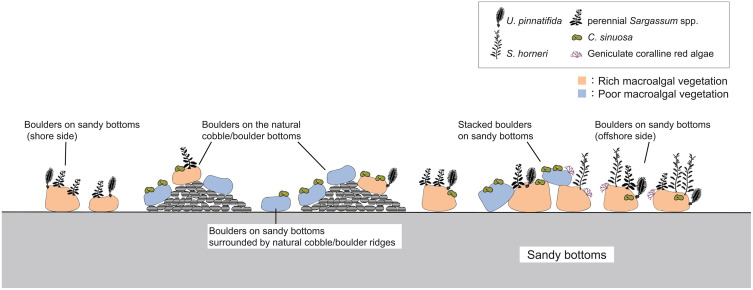
Summary of the macroalgal distribution on the man-made boulders reef. *U*. *pinnatifida* and perennial *Sargassum* spp. were present on the boulders on the sandy bottom and on the shore side. On the boulders on the natural cobble/boulder bottom, *C*. *sinuosa* communities and bare rock dominated, and a few perennial *Sargassum* spp. and *U*. *pinnatifida* present on the high relative height or offshore side near the sandy bottoms. In areas where the boulders on the sandy bottoms were surrounded by natural cobble/boulder ridges, the *C*. *sinuosa* community was dominant. On the boulders on the sandy bottom and offshore sides, a rich macroalgal community composed of various seaweed species was observed. *S*. *horneri* and geniculate coralline red algae were present only on the offshore side. On some piled boulders, poor vegetation was observed.

Based on the results of this study, the boulders should not be installed on natural cobble/boulder reefs for efficient macroalgal beds creation. Installing boulders at low density on sandy bottoms is efficient for macroalgal bed creations (Fig 8), this may be due to low density of sea urchins. Boulders placed on the natural cobble reefs provide habitat for the purple sea urchin *H*. *crassispina*, potentially leading to greater seaweed decline. Even on sandy bottoms, locations enclosed by cobble bottom and piled up boulders may also provide a habitat for *H*. *crassispina*.

At the study site, 561 artificial boulders (half of the total) were placed on natural cobble/boulder reefs (Fig 3). If all the artificial boulders were located only on the sandy bottoms, twice the area of the macroalgal beds could have been created, or the same amount of macroalgal beds could have been created using half the number of artificial boulders. Installing boulders or artificial structures on sandy bottom can disturb the existing habitat of organisms that depend on sandy bottom [115,116]. This can interfere with organisms that live buried in the sediment. In addition, more fine sediments are accumulated by installing on artificial structures on soft bottoms [117,118]. Additionally, arrival of biota that prefer hard substrates can alter the species and abundance of predators and prey. Therefore, appropriate site selection is important to create macroalgal beds effectively and minimize seafloor modification.

With rising seawater temperature in the near future, it is uncertain whether the sandy bottoms remain suitable for establishments of macroalgal beds. Unlike the purple sea urchin *H*. *crassispina*, the dominant species at the study site, the long-spined sea urchin *Diadema setosum*—a subtropical species—also inhabits substrates surrounded by sandy bottoms [38,119,120]. In addition, grazing by herbivorous fish was observed regardless of the bottom type [119,121]. Furthermore, increased feeding activity due to enhanced metabolic rates under higher temperatures [122], and the possible arrival of tropical herbivorous species [35] could further intensify grazing pressure. Consequently, the artificial macroalgal beds established on the sandy bottoms at the study site may also decline in the near future as well as that on the cobble/boulder bottoms.

The universal applicability of these suggestions remains uncertain. Suitable habitat conditions for establishments of macroalgal beds may vary depending on the location of broader scale, regions or seaweed species, so site selection for macroalgal beds restoration must be examined accordingly. Understanding the commonalities and differences in seaweeds distribution across region and taxonomic groups is important for grasping the factors involved in the establishment of seafloor vegetation, seascape, and ecosystems.

### 4.3 Advantages of underwater photogrammetry

Underwater photogrammetry survey can obtain many information such as seafloor topography, seafloor vegetation, bottom type, and geomorphic indicators with low effort and low cost. In this study, more than 2,000 m^2^ of seafloor topography was reconstructed within just one hour of SCUBA diving by a single diver. Identification of seaweed species and vegetation communities is the advantage of underwater photogrammetry, compared to the methods such as UAV, side-scan sonar or multibeam sonar. From the high-resolution 3D model of the seafloor topography, various geomorphic indicators can be computed, such as relative height, surface complexity, if necessary, slope and orientation, which were difficult to measure directly in the limited time available for SCUBA diving. This enabled us to quantitatively elucidate habitat suitable conditions for benthic organisms.

It should be noted that seafloor vegetation mapping by underwater photogrammetry can be applied in the macroalgal beds with low to medium coverage, that most of the seafloor surface is visible. In the case seaweeds cover the entire seafloor and the seafloor surface is invisible, underwater photogrammetry cannot be applied. Seasonal macroalgal beds with the season most of seaweed species are starting to grow, are suitable for photogrammetry vegetation mapping.

The high-resolution seafloor vegetation map made in this study allowed us to visually understand suitable and unsuitable environments for establishing macroalgal beds. This can be shared with fishery cooperatives or coastal developers who may not have access to underwater observations. Seafloor maps created by photogrammetry contribute to the dissemination of scientific knowledge to society.

In this study, only the macroalgal vegetation was examined in the presence/absence of each seaweed species. More detailed 3D models using higher resolution photographs may allow us to investigate the effect of the boulder size or shape on seafloor vegetation and to quantify the coverage of each species. This underwater photogrammetry survey can be applied to investigate patterns of habitat use (e.g., spawning, feeding, and territorial preferences) by fish and invertebrates, not just macroalgal vegetation.

## 5 Conclusion

High-resolution seafloor vegetation mapping using underwater photogrammetry enabled us to quantify and visualize suitable and unsuitable environmental conditions for establishing macroalgal beds. For the efficient macroalgal beds creation, installing boulders at low density on the sandy bottoms would be better, while avoiding areas on or near natural cobble/boulder reefs, is recommended. When restoring macroalgal beds, it is necessary to consider suitable conditions for establishing macroalgal beds and future environmental changes to avoid producing barren areas and exacerbating environmental degradation. The method developed in this study is expected to aid in site selections for macroalgal beds creation, artificial reef design, and improvement of restoration techniques for seafloor ecosystems. High resolution mapping of the seafloor vegetation and seascape is important for proper managements of seafloor ecosystems.

## Supporting information

S1 Text Procedures for the 3D model modification of scale, orientation and depth. (DOCX)

S2 Fig Species of seaweeds present on the artificial boulders in the survey area. (a) *Undaria pinnatifida*, (b) *Sargassum horneri*, (c) *S*. *patens*, one of the perennial *Sargassum* (d) *Colpomenia sinuosa*, (e) geniculate coralline red algae.
(TIF)

S3 Fig Geomorphic indicators: relative height and surface complexity computed on the 3D model. Values of geomorphic indicators are excluded in outer edge areas of the 3D model where there are not enough surrounding vertices.
(TIF)

S4 Fig Schematic diagram of relative height calculation in the case of two-dimension. Relative height was determined as the height from the deepest point within a radius of 1.5 meters. Snapshots from the 3D model (b, c) show the piled boulders with high complexity (b) and an isolated boulder with low complexity (c).
(TIF)

S5 Text Essential part of source code for calculating relative height made by Julia language 1.5.3. Processing for boundaries and fast computation techniques was omitted. The descriptions after # are comment-outs.
(DOCX)

S6 Fig Schematic diagram of surface complexity calculation in the case of two-dimension. Surface complexity was calculated as follows in the case of two-dimensional: Consider a square with a side length of 3.0 meters around a target vertex, and divide this square into a grid with a side length of 0.5 meters. Then, count the overlaid grid cells that overlap with the vertices (gray filled squares), similar to the box counting method. The percentage of counts relative to the total grid counts (in the case of two-dimensional, 6^2^ = 36) for this squared is referred to as surface complexity. In this case, the count is 9, and surface complexity calculated as 9/36 = 0.25.
(TIF)

S7 Text Essential part of source code calculating surface complexity made by Julia language 1.5.3. Processing for boundaries and fast computation techniques was omitted. The descriptions after # are comment-outs.
(DOCX)

S8 Data Dataset. Dataset of vegetation type, bottom type, and geomorphic indicators (relative height and surface complexity) for all artificial boulders at the study site. In the columns from “*U*. *pinnatifida*” to “Geniculate coralline red algae”, “+” indicates that each species was present and “−” indicates that it was absent. In the “Bottom type” column, “sand” and “cobble” indicate each boulder on sandy bottom and on natural cobble/boulder bottom. The “Vegetation type” column shows the seafloor vegetation type, which is classified according to Table 2. In the “Relative Height” and “Surface Complexity” columns, geomorphic indicators (relative height and surface complexity) were shown. For the 18 boulders located in outer edge areas of the 3D model, geomorphic indicators were not calculated (S3 Fig).
(CSV)

S9 Fig The purple sea urchin *Heliocidaris crassispina* in Himeshima Island. (a) Aggregation of *H*. *crassispina* in Februrary. (b) In June, few sea urchins were observed at the same reef.
(TIF)
