# Supplementary material for: Seafloor vegetation map of man-made boulders reef by underwater photogrammetry: Suggestions for site selections in macroalgal bed creations
Source: PLoS One. 2026 Mar 2;21(3):e0341865. doi: 10.1371/journal.pone.0341865 (PMC12952637; doi:10.1371/journal.pone.0341865)
Supplement: S1 Text — (DOCX) [file pone.0341865.s001.docx]

The resulting 3D model reconstructed by Metashape consisted of 85,074,129 faces and 42,546,171 vertices, which was too large for the subsequent geomorphic indicator computations. The 3D model was reduced to 785,063 vertices and 1,566,903 faces using the *Decimate Mesh* tool, and then exported in PLY format.

Modification of the obtained 3D model was used by Meshlab and Julia language 1.5.3 (Fig. 2c). Parts of the 3D model where topography was not accurately reconstructed were removed. During the underwater photographing survey, two points exactly 1.0 m apart from each point were recorded using a 1 m square frame, along with the north-south axis. The scale of the 3D model was adjusted so that 1.0 in the 3D model’s coordinate system corresponds to 1.0 m using the *Transform: Scale, Normalize* tool in Meshlab. The north-south axis was adjusted to coincide with the y-axis in the 3D model using the *Transform: Rotate* tool in Meshlab.

Baseline depth values were measured at the three reference points (Fig. 2b) using AQUALUNG CALM Plus+ dive computer. First, the coordinates of the 3D model were adjusted by parallel translation to match the baseline depth with one of the reference points using the tool *Transform: Translate, Center, set Origin* tool in Meshlab. Then, a rotation matrix *R* was computed based on the depth data and orientation, and this matrix *R* was multiplied with coordinates of all vertices in the 3D model to compute the depth adjustment.
