# Supplementary figures and images for: Seafloor vegetation map of man-made boulders reef by underwater photogrammetry: Suggestions for site selections in macroalgal bed creations
Source: PLoS One. 2026 Mar 2;21(3):e0341865. doi: 10.1371/journal.pone.0341865 (PMC12952637; doi:10.1371/journal.pone.0341865)

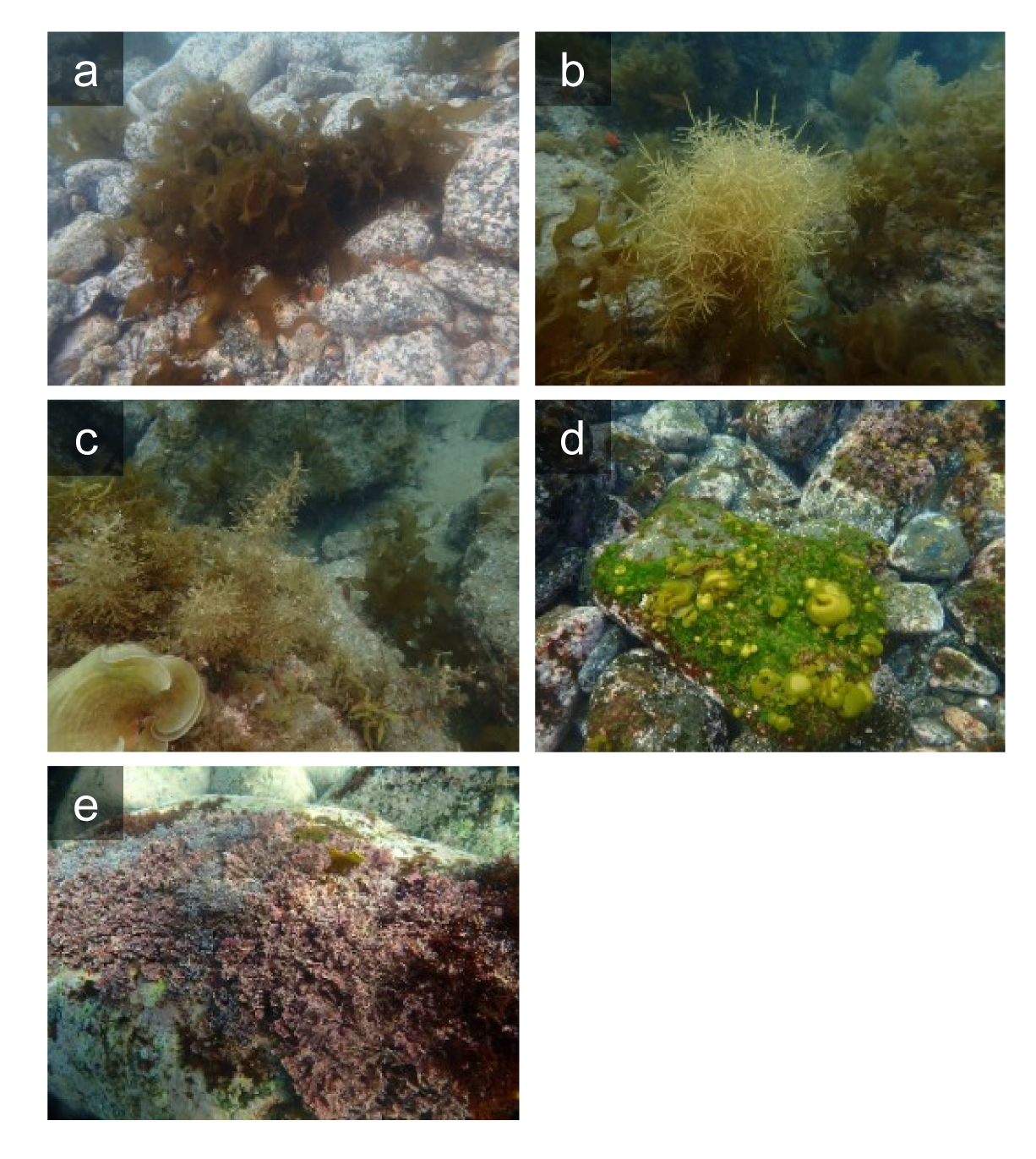

Supplement: S2 Fig — (a) Undaria pinnatifida, (b) Sargassum horneri, (c) S. patens, one of the perennial Sargassum (d) Colpomenia sinuosa, (e) geniculate coralline red algae. (TIF) [file pone.0341865.s002.tif]

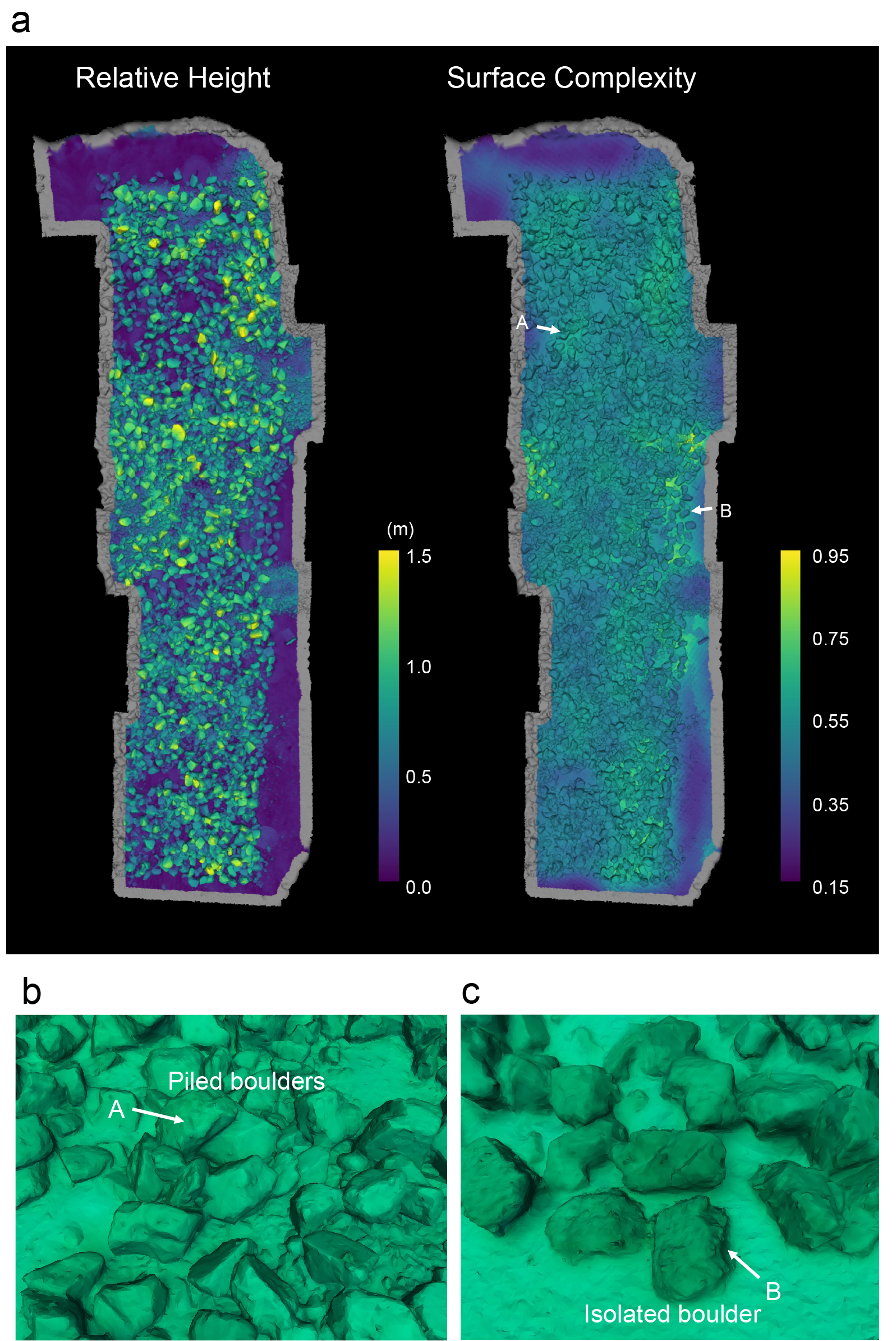

Supplement: S3 Fig — Values of geomorphic indicators are excluded in outer edge areas of the 3D model where there are not enough surrounding vertices. (TIF) [file pone.0341865.s003.tif]

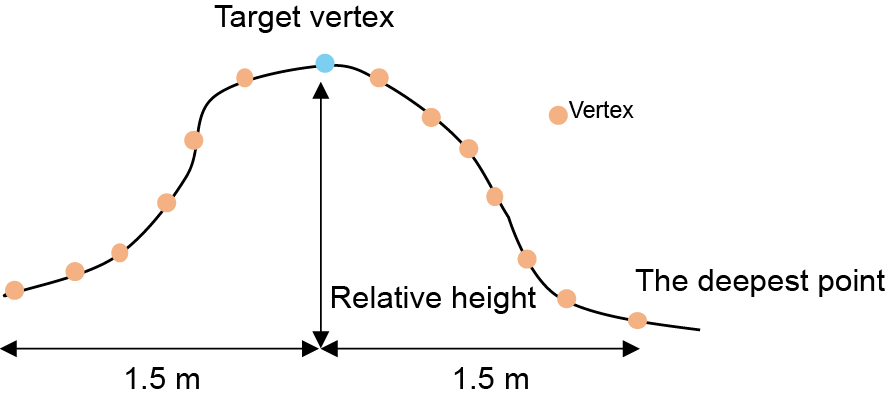

Supplement: S4 Fig — Relative height was determined as the height from the deepest point within a radius of 1.5 meters. Snapshots from the 3D model (b, c) show the piled boulders with high complexity (b) and an isolated boulder with low complexity (c). (TIF) [file pone.0341865.s004.tif]

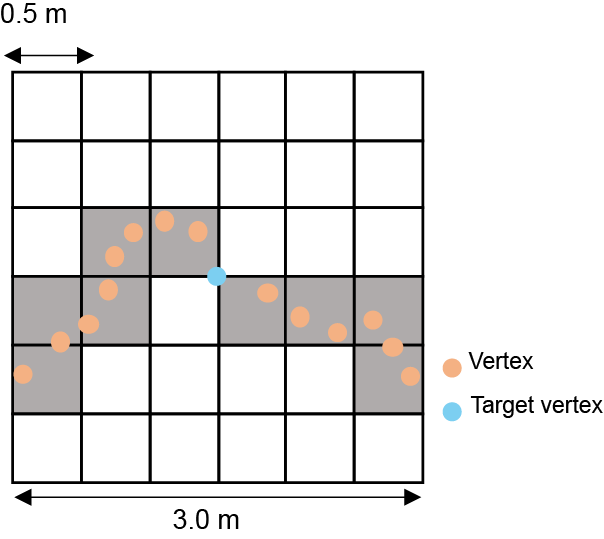

Supplement: S6 Fig — Surface complexity was calculated as follows in the case of two-dimensional: Consider a square with a side length of 3.0 meters around a target vertex, and divide this square into a grid with a side length of 0.5 meters. Then, count the overlaid grid cells that overlap with the vertices (gray filled squares), similar to the box counting method. The percentage of counts relative to the total grid counts (in the case of two-dimensional, 62 = 36) for this squared is referred to as surface complexity. In this case, the count is 9, and surface complexity calculated as 9/36 = 0.25. (TIF) [file pone.0341865.s006.tif]

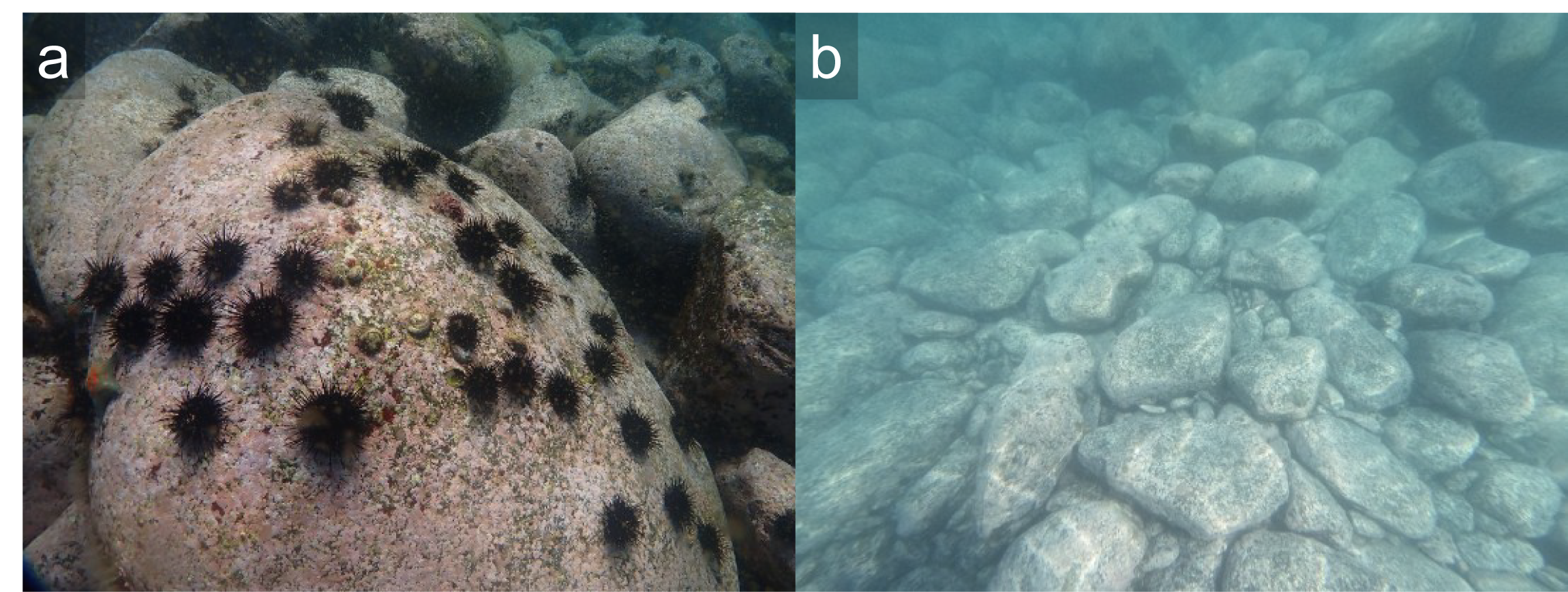

Supplement: S9 Fig — (a) Aggregation of H. crassispina in Februrary. (b) In June, few sea urchins were observed at the same reef. (TIF) [file pone.0341865.s009.tif]
