# Supplementary material for: Seafloor vegetation map of man-made boulders reef by underwater photogrammetry: Suggestions for site selections in macroalgal bed creations
Source: PLoS One. 2026 Mar 2;21(3):e0341865. doi: 10.1371/journal.pone.0341865 (PMC12952637; doi:10.1371/journal.pone.0341865)
Supplement: S5 Text — Processing for boundaries and fast computation techniques was omitted. The descriptions after # are comment-outs. (DOCX) [file pone.0341865.s005.docx]

**S5 Text**. **Essential part of source code for calculating relative height made by Julia language 1.5.3.** Processing for boundaries and fast computation techniques was omitted. The descriptions after # are comment-outs.

| #Relative Height  #v_xyz: xyz coordinates of 3D model vertices (The x-axis direction is north, y-axis is east, and z-axis is vertical upward. “size(v_xyz)[1]” in source code means the number of vertices, 785603.  # A case of computing relative height on all vertices of the 3D model.  RelativeHeight = zeros(size(v_xyz[1])  for i in 1:size(v_xyz)[1]  #computing Euclidean distances from a target vertex ($v_{i}$) on the all vertices of the 3D model  Euclid_distances = sqrt. (sum((v_xyz .- reshape(v_xyz[i,:], 1:3)).^2, dims=2))  #Vertices whose Euclidean distances from $v_{i}$ are less than 1.5 meter as neighboring vertices.  neighbor_index = findall (Euclid_distances [:, 1] .< 1.5)  #Relative height was calculated as:  RelativeHeight [i] = v_xyz[i, 3] - findmin(v_xyz[neighbor_index, 3]) [1]  End |
| --- |
