# Supplementary material for: Seafloor vegetation map of man-made boulders reef by underwater photogrammetry: Suggestions for site selections in macroalgal bed creations
Source: PLoS One. 2026 Mar 2;21(3):e0341865. doi: 10.1371/journal.pone.0341865 (PMC12952637; doi:10.1371/journal.pone.0341865)
Supplement: S7 Text — Processing for boundaries and fast computation techniques was omitted. The descriptions after # are comment-outs. (DOCX) [file pone.0341865.s007.docx]

**S7 Text**. **Essential part of source code calculating surface complexity made by Julia language 1.5.3.** Processing for boundaries and fast computation techniques was omitted. The descriptions after # are comment-outs.

| #Surface Complexity  #v_xyz: xyz coordinates of 3D model vertices  # A case of computing surface complexity on all vertices of the 3D model.  cell_size = 0.50 #Size of 3D cells  cube_width = 3.0 #Range of box counting  #Step. 1: converting vertices of a 3D model to a 3D raster model with 0.50 m resolution (*cell_size*)  min_x, min_y, min_z = findmin(v_xyz, dims=1)[1]  max_x, max_y, max_z = findmax(v_xyz, dims=1)[1]  x_grid_num = Int64(floor((max_x - min_x) / cell_size) + 1)  y_grid_num = Int64(floor((max_y - min_y) / cell_size) + 1)  z_grid_num = Int64(floor((max_z - min_z) / cell_size) + 1)  Raster_model = zeros (Bool, x_grid_num, y_grid_num, z_grid_num)  for i in 1:size(v_xyz)[1]  x_i=Int64(floor((v_xyz[i, 1] - min_x) / cell_size) + 1)  y_i=Int64(floor((v_xyz[i, 2] - min_y) / cell_size) + 1)  z_i=Int64(floor((v_xyz[i, 3] - min_z) / cell_size) + 1)  Raster _model [x_i, y_i, z_i] = 1  end  #Step. 2: Counting non-zero grid cells within the cube of side length *cube_length* around a target vertex.  Surface_complexity= zeros(size(v_xyz)[1])  for i in 1: size(v_xyz)[1]  x1 = Int64(floor((v_xyz[i, 1] - min_x) / cell_size + 1) – (cube_length / 2) / cell_size)  x2 = Int64(floor((v_xyz[i, 1] - min_x) / cell_size + 1) + (cube_length / 2) / cell_size)  y1 = Int64(floor((v_xyz[i, 2] - min_y) / cell_size + 1) – (cube_length / 2) / cell_size)  y2 = Int64(floor((v_xyz[i, 2] - min_y) / cell_size + 1) + (cube_length / 2) / cell_size)  z1 = Int64(floor((v_xyz[i, 3] - min_z) / cell_size + 1) – (cube_length / 2) / cell_size)  z2 = Int64(floor((v_xyz[i, 3] - min_z) / cell_size + 1) + (cube_length / 2) / cell_size)  Surface_complexity [i]=sum(Grid_model [x1:x2, y1:y2, z1:z2])/(cube_length / cell_size)^3  end |
| --- |
